# Fenfluramine for the treatment of status epilepticus: use in an adult with Lennox–Gastaut syndrome and literature review

**DOI:** 10.1186/s42466-023-00306-z

**Published:** 2024-02-22

**Authors:** Adam Strzelczyk, Hendrik Becker, Lisa Tako, Susanna Hock, Elke Hattingen, Felix Rosenow, Catrin Mann

**Affiliations:** 1 Goethe-University Frankfurt, Epilepsy Center Frankfurt Rhine-Main, Department of Neurology, University Hospital Frankfurt, Schleusenweg 2-16 (Haus 95), 60528 Frankfurt am Main, Germany; 2 Goethe-University Frankfurt, Department of Neuroradiology, University Hospital Frankfurt, Frankfurt am Main, Germany

**Keywords:** Epilepsy, Seizure, Dravet, Anticonvulsants, Encephalopathy

## Abstract

**Background:**

Novel treatments are needed to control refractory status epilepticus (SE). This study aimed to assess the potential effectiveness of fenfluramine (FFA) as an acute treatment option for SE. We present a summary of clinical cases where oral FFA was used in SE.

**Methods:**

A case of an adult patient with Lennox–Gastaut syndrome (LGS) who was treated with FFA due to refractory SE is presented in detail. To identify studies that evaluated the use of FFA in SE, we performed a systematic literature search.

**Results:**

Four case reports on the acute treatment with FFA of SE in children and adults with Dravet syndrome (DS) and LGS were available. We report in detail a 30-year-old woman with LGS of structural etiology, who presented with generalized tonic and dialeptic seizures manifesting at high frequencies without a return to clinical baseline constituting the diagnosis of SE. Treatment with anti-seizure medications up to lacosamide 600 mg/d, brivaracetam 300 mg/d, valproate 1,600 mg/d, and various benzodiazepines did not resolve the SE. Due to ongoing refractory SE and following an unremarkable echocardiography, treatment was initiated with FFA, with an initial dose of 10 mg/d (0.22 mg/kg body weight [bw]) and fast up-titration to 26 mg/d (0.58 mg/kg bw) within 10 days. Subsequently, the patient experienced a resolution of SE within 4 days, accompanied by a notable improvement in clinical presentation and regaining her mobility, walking with the assistance of physiotherapists. In the three cases reported in the literature, DS patients with SE were treated with FFA, and a cessation of SE was observed within a few days. No treatment-emergent adverse events were observed during FFA treatment in any of the four cases.

**Conclusions:**

Based on the reported cases, FFA might be a promising option for the acute treatment of SE in patients with DS and LGS. Observational data show a decreased SE frequency while on FFA, suggesting a potentially preventive role of FFA in these populations.

**Key points:**

- We summarize four cases of refractory status epilepticus (SE) successfully treated with fenfluramine.
- Refractory SE resolved after 4–7 days on fenfluramine.
- Swift fenfluramine up-titration was well-tolerated during SE treatment.
- Treatment-emergent adverse events on fenfluramine were not observed.
- Fenfluramine might be a valuable acute treatment option for SE in Dravet and Lennox–Gastaut syndromes.

## Background

Refractory status epilepticus (SE) is characterized by unresponsiveness to initial therapy with benzodiazepines and anti-seizure medications (ASMs). It occurs in approximately 40–50% of all cases of SE [1]. Status epilepticus is associated with many fatalities and high morbidity [2–4], and treatment is difficult in refractory cases due to the failure of first- and second-line therapies. Therapeutic management beyond the use of benzodiazepines and common ASMs is based on clinical reports, expert opinion, and pathophysiological assumptions from experimental data. Patients with developmental and epileptic encephalopathies (DEEs) such as Dravet syndrome (DS) and Lennox–Gastaut syndrome (LGS) frequently experience convulsive and non-convulsive SE [5, 6], and their treatment is more challenging because some ASMs may exacerbate seizures and SE, including sodium channel blockers in DS [7, 8].

Recently, fenfluramine (FFA) was licensed in Europe and the US as an adjunctive treatment for epileptic seizures in DS and LGS patients over 2 years of age. FFA is derived from amphetamine and has different modes of action than other ASMs. FFA modulates serotonergic neurotransmission and thus produces an overall increase in serotonin levels in the synaptic cleft by increasing central serotonin release and simultaneously reducing presynaptic serotonin reuptake [9, 10]. FFA acts directly on some specific serotonin receptors (HTR) including 5-HT1D, 5-HT2A, and 5-HT2C receptors through its main metabolite, norfenfluramine. It additionally seems to positively modulate the sigma-1 receptor, which belongs to a group of chaperone proteins. On activation, the sigma-1 receptor modulates various voltage-gated ion channels (Ca2 +, Na +, and K +) and NMDA receptors, which influence excitatory and inhibitory neurotransmission [9, 10]. The efficacy of FFA has been demonstrated in four double-blind, placebo-controlled, randomized phase III studies in DS and LGS patients [11–14]. Details on the use of FFA and pharmacological properties are presented in Table 1 [15].

**Table 1:** Clinical use of fenfluramine and its pharmacological properties

Drug name	Fenfluramine
Chemical structure	
Indication	Add-on treatment in Dravet and Lennox-Gastaut syndrom from 2 years of age
Mode of action	Modulates serotonergic neurotransmission Acts directly on some specific serotonin receptors (HTR) including 5-HT1D, 5-HT2A, and 5-HT2C receptors Positively modulates the sigma-1 receptor
Route of administration	Oral solution
Pharmacokinetics	Fat-soluble, high bioavailability not influenced by nutrition Steady state usually reached after 4 days Plasma-elimination half-life of 20 h Primarily renal elimination
Dose regimen	Start at 0.1 mg/kg twice daily (0.2 mg/kg/body weight (bw)/day), increase to 0.2 mg/kg twice daily (0.4 mg/kg bw/day) after 7 days, and to a maximum of 2 × 0.35 mg/kg daily (0.7 mg/kg bw/day) after a further 7 days Slower uptitration in an outpatient setting of 0.1 mg/kg bw or 1 ml per week improves tolerability, faster uptitration in emergency situations is possible. The maximum total daily dose of 26 mg FFA or 0.7 mg/kg bw must not be exceeded (17 mg FFA or 0.4 mg/kg bw if stiripentol is coadministered).
Typical adverse events	Decreased appetite, loss of weight, fatigue, somnolence
Precautions	Prior to FFA, patients must undergo an echocardiogram to exclude valvular heart disease or pulmonary hypertension. Echocardiogram monitoring should be conducted every 6 months for the first 2 years and annually thereafter.
Pivotal trials	Lagae et 2020; Nabbout et al. 2020; Knupp et al. 2022; Sullivan et al. 2023 [11–14]

bw: body weight; chemical structure derived from [16]

Given the severity of illness and unfavorable outcomes in patients with refractory SE, therapies with different modes of action are critical to stopping ongoing seizure activity. The objective of this study was to determine the use, efficacy, and tolerability of FFA in patients with refractory SE.

## Methods

Since the availability of FFA in our department (early 2020 within the Early Access Program), we recorded in detail all patients treated with it. Between April 2020 and November 2023, FFA was started de novo in one adult patient during refractory SE. CARE guidelines for reporting case reports were followed [17].

To identify further studies evaluating the use of FFA in SE, we performed a systematic literature search in the MEDLINE, Cochrane Central Register of Controlled Trials, and Excerpta Medica databases from 1996 until November 2023, using a combined search strategy including the following keywords: “fenfluramine”, “epilepsy”, “seizure”, “Dravet”, “Lennox–Gastaut”, “epileptic encephalopathy”, and “status epilepticus”. We examined the reference lists of all identified studies and review articles on FFA [9, 18] for additional relevant studies and reports.

The classifications of seizure type, epilepsy type and syndrome, and SE were adopted based on the latest definitions proposed by the International League Against Epilepsy (ILAE) [19–21]. Regarding seizure duration, the ILAE SE definition was applied and considered all tonic–clonic seizures lasting for more than 5 min, as well as focal seizures with impaired consciousness and absence seizures lasting for more than 10 min [21]. Refractory SE refers to recurrent seizure activity notwithstanding the administration of two ASMs appropriately selected and dosed, including a benzodiazepine [22, 23].

The data collected included demographics, clinical diagnosis, etiology, semiology, history of seizures or SE, total length of stay in hospital, modified Rankin Scale (mRS), and Status Epilepticus Severity Score [24] on admission. The cessation of SE and further seizure freedom were the primary outcome measures used to demonstrate ASM benefits. Treatment was considered successful when no further ASMs were administered until cessation of SE.

Regarding the efficacy of FFA, the duration of SE before the initiation of FFA and the number of ASMs used before FFA initiation were analyzed. The timing of FFA with respect to SE onset and cessation and the presence of adverse events were also collected.

The maximum recommended daily dose of FFA for the treatment of DS or LGS is 0.7 mg per kg bodyweight or a total maximum of 26 mg (0.4 mg/kg bw/day and a total of 17 mg maximum daily dose in comedication with stiripentol), divided into two doses during the day. The recommended treatment initiation is 0.2 mg per kg body weight per day and a gradual increase over 2 weeks to the maximum dose [25].

## Results

### Case presentation

This 30-year-old woman had LGS with tonic (associated with falls), generalized tonic–clonic, and focal impaired awareness seizures with gaze deviation to the right side, mostly occurring as clusters of four to six seizures over a few days every 4–6 weeks since the age of 6 months. Furthermore, at the age of 27 years, two episodes of SE occurred: one associated with infection and another with an undetermined cause. Febrile seizures were not reported during childhood. Genetic testing for DEEs including SCN1A, SCN1A (MLPA), PCDH19, SCN2A, CHD2, GABRA1, STXBP1, and HCN1 was negative [26]. Initial imaging studies did not show an underlying structural cause of the epilepsy. Besides seizures, autism spectrum disorder, severe communication impairment, ataxia, and mild tetraparesis were present since childhood. The patient maintained mobility and was able to walk a few kilometers. No seizure remission had been achieved on various combinations of ASMs.

At the age of 30, the patient presented with generalized tonic and focal impaired awareness seizures manifesting at high frequencies without a return to clinical baseline, constituting the diagnosis of SE. On admission, the ASM regimen consisted of lacosamide 350 mg/d, brivaracetam 100 mg/d, and clonazepam 0.75 mg/d. No clinical or laboratory hints of infection existed. The EEG showed generalized slowing, focal and generalized sharp waves, and 11 seizure patterns with generalized paroxysmal fast activity, coinciding with clinical manifestations of a tonic seizure within 20 min of recording.

**Fig. 1:**
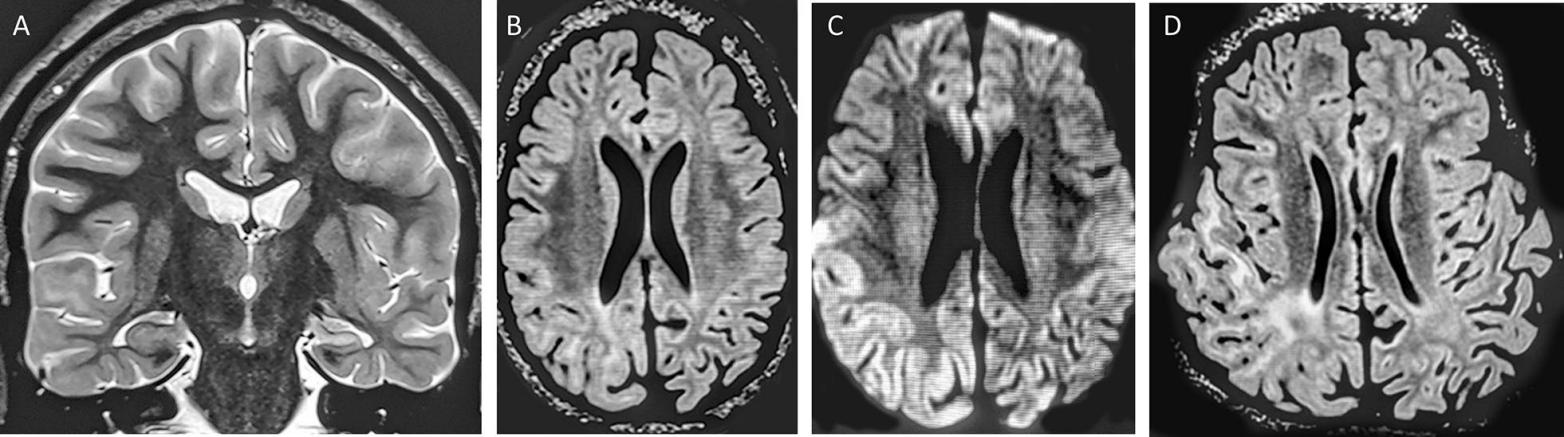
Etiology of Lennox–Gastaut syndrome was assumed due to various structural pathologies. Coronal PD-weighted image shows hippocampal sclerosis and atrophy of the temporal lobe, whereas the right superior temporal gyrus is swollen and white matter is hyperintense (**A**). The axial FLAIR (**B**) and corresponding DWI (**C**) show ictal swelling of the right parietal gyri with white matter edema and cortical diffusion restriction, while the contralateral gyri in the left hemisphere are atrophic. Coronal surface reformatting of the 3D FLAIR (**D**) shows how the ictal changes extend across the temporoparietal brain region. See again atrophy in the left hemisphere

Subsequent adjustments to the ASM regimen, with increases of brivaracetam to 300 mg/d and lacosamide to 600 mg/d, a switch to lorazepam 2 mg/d, and the introduction of valproate 1,600 mg/d, did not result in a resolution of SE. Due to elevated ammonia levels, valproate had to be reduced to 800 mg/d, and lorazepam was replaced by clobazam 15 mg/d. The patient’s clinical course was complicated by a urinary tract infection. Seizure and SE were recorded on continuous EEG monitoring. A brain MRI showed various pathologies corresponding with a structural etiology of LGS, while FLAIR and DWI show ictal changes in the right temporoparietal region (Fig. 1).

Due to ongoing refractory SE and following an unremarkable echocardiography, treatment was initiated with FFA, with an initial dose of 10 mg/d (0.22 mg/kg bw), and fast up-titration to 26 mg/d (0.58 mg/kg bw) within 10 days. FFA solution (Fintepla® 2.2 mg/ml, UCB Pharma S.A., Bruxelles, Belgium) was given through a nasogastric tube twice per day.

No adverse events were observed. Subsequently, the patient experienced a resolution of SE within 4 days, accompanied by a notable improvement in clinical presentation and regaining her mobility, walking with the assistance of physiotherapists. Valproate could be withdrawn from the therapy after SE cessation, and the patient was discharged with brivaracetam 300 mg/d, lacosamide 400 mg/d, clonazepam 0.5 mg/d, and fenfluramine 26 mg/d.

At 2 months follow-up, the caregiver reported an almost seizure-free outcome, with only one tonic seizure since discharge. Mobility improved, with a walking distance of 500 to 1,000 m. The caregivers reduced ASMs to brivaracetam 200 mg/d, lacosamide 400 mg/d, and fenfluramine 17.6 mg/d, while clonazepam has been discontinued.

### Literature review

Three additional cases of treatment of SE with FFA were identified and are summarized along with the current case in Table 2. The first report, published by Specchio et al., was on the treatment of an 8-year-old boy with DS in whom FFA was started in a non-convulsive SE [27]. Trowbridge et al. reported on a 5-year-old boy with frequent SE episodes in whom FFA stopped the series of SE [28]. The first adult with DS treated with FFA during a super-refractory SE was reported by Millet et al. [29]. The number of prior failed ASMs for the treatment of SE before FFA was started ranged between 2 and 14, the SE was resolved in EEG within 4–7 days following the start of FFA. Follow-up data were reported for periods between 1 month and 2 years and 10 months. In all cases, FFA was maintained in the long term.

**Table 2:** Clinical characteristics, management, and outcome of patients treated with fenfluramine for status epilepticus

Data	Adult with LGS, current report	Adult with DS, Millet et al., 2021	Child with DS, Specchio et al., 2020	Child with DS, Trowbridge et al., 2021
Age (y)	30	20	8	5
Sex	Female	Female	Male	Male
mRS before admission	3	4	Not reported	Not reported
Awareness at admission	Stuporous	Stuporous	Stuporous	Not reported
Diagnosis/etiology	Lennox–Gastaut syndrome, structural etiology	Dravet syndrome, *SCN1A* frameshift mutation (two base-pair [AT] insertion at 3725–3726)	Dravet syndrome, c.664 C > T (p.R222X) *SCN1A* variant	Dravet syndrome, c.4162G > T (p.Glu1388Ter) *SCN1A* variant
SE semiology	NCSE with frequent tonic seizures	Tonic SE	NCSE with frequent tonic and bilateral tonic–clonic seizures	Recurrent, frequent SE (both NSCE and CSE); FFA start in interval
STESS score	3	3	3	Not reported
ASM therapy before admission	LCM 350 mg/d BRV 100 mg/d CZP 0.75 mg/d	LEV 2,500 mg/d VPA 2,000 mg/d CLB 20 mg/d KD	VPA 28.3 mg/kg/d CLB 0.6 mg/kg bw/d	LEV, CZP, CLB, VPA, KD
Therapy with maximal dosage of ASMs/24 h *co-administered with FFA	1.) LZP 4 mg/d 2.) VPA 1,500 mg/d 2.) LCM 600 mg/d 2.) BRV 300 mg/d 3.) CLB 15 mg/d 4.) FFA 26 mg/d (0.58 mg/kg bw)	1.) LZP 4 mg/d 1.) Propofol 100 µg/kg/min 1.) VPA 9,000 mg/d 1.) CLB 30 mg/d 1.) LEV 6,000 mg/d 1.) VNS; KD 2.) MDZ 30 mg/hr 3.) Pentobarbital 2.5 mg/kg/hr 4.) CBD 20 mg/kg bw/d 5.) Felbamate 1,800 mg/d 6.) Ketamine 6 mg/kg/hr 7.) PB 200 mg/d 8.) BRV 300 mg/d 9.) FFA 0.7 mg/kg bw/d	Unclear; VPA and CLB FFA 26 mg/d* (0.52 mg/kg bw/d*)	FFA 0.7 mg/kg bw/d VPA LEV CLB KD
Duration of SE before FFA	16 d	36 d (0.4 mg/kg bw/d) 42 d (0.7 mg/kg bw/d)	Unclear	No further episode of SE since initiation of FFA
Time to EEG resolution after FFA	4 d	7d (post 0.7 mg/kg/d)	4 d	Not reported
Concurrent ASMs with FFA	BRV; CLB; LCM, VPA	BRV; VPA; CLB; PB; ketamine; KD, VNS	Not reported	LEV, VPA, CLB, FFA
Ventilation time	0	55 d	Not reported	Not applicable
Total length of stay	37 d	56 d	Not reported	Not applicable
mRS score at discharge	4	4	Not reported	Not applicable
Disposition	Home	Rehabilitation facility	Not reported	Not applicable
Follow-up	2 months	2 years 3 months	1 month	2 years 10 months
mRS at last follow-up	3	Not reported	Not reported	Not reported
ASMs at follow-up	FFA 17.6 mg/d, BRV 200 mg/d, LCM 400 mg/d	FFA 0.7 mg/kg bw/d; VPA 4,000 mg/d; BRV 200 mg/d; PB 194.4 mg/d; CLB 40 mg/d; VNS; KD	FFA 0.6 mg/kg bw/d; VPA; CLB	LEV, VPA, CLB, FFA

ASM: anti-seizure medication; bw: body weight; BRV: brivaracetam; CBD: cannabidiol, CLB: clobazam; CZP: clonazepam; FFA: fenfluramine; KD: ketogenic diet; LEV: levetiracetam; LZP: lorazepam; MDZ: midazolam; mRS: modified Rankin Scale;; NCSE: non-convulsive status epilepticus; PB: phenobarbital; SE: status epilepticus; STESS: Status Epilepticus Severity Score; TPM: topiramate; VNS: vagal nerve stimulator; VPA: valproate

*reported as 30 mg/d 0.6 mg/kg/d FFA hydrochloride

## Discussion

We present data on four patients who received acute treatment with FFA for SE. In all cases, a resolution of SE within a few days after starting administration of FFA was observed, and no treatment-emergent adverse events were reported. Although we describe a limited case series of patients, the data suggest that FFA may be a useful and well-tolerated acute therapeutic approach in treating refractory SE in patients with DS and LGS.

We describe an adult who fulfilled the diagnostic criteria for LGS [30], and the MRI revealed structural changes as underlying cause. Despite a prolonged treatment course of SE, the patient returned to baseline functioning and achieved near seizure freedom at the last follow-up, and the ASM drug load could be reduced. The other three cases with DS reported stable ASM drug regimens. A more extended follow-up would be desirable in all cases.

In addition to these four case reports, several publications have demonstrated a diminished incidence of SE during treatment with FFA in real-world settings. A German multicenter analysis of 78 DS patients treated in the Early Access Programme showed a significantly decreased number of SE episodes, from 28% of patients in the 6 months before FFA to only 14% during a 6-month follow-up while on FFA. In addition, 45% of patients were able to discontinue concomitant ASMs, and a further 23% reduced their intake. The retention rate was 92% in adults and 83% in children [31].

Zhu et al. reported the successful treatment of an 8-month-old boy diagnosed with early infantile epileptic encephalopathy (EIEE), Dravet-like syndrome, and global developmental delays due to biallelic *SCN1B* genetic variants [32]. Seizures began at the age of 3 weeks, and by the age of 2 months, myoclonic twitching had evolved to nearly continuous myoclonic seizures. By the age of 4 months, the patient repeatedly presented with convulsive SE and responded only for a short time to multiple ASMs at high doses. Pyridoxine, topiramate, clobazam, and a ketogenic diet also failed. At the age of 8 months, FFA titrated up to 0.7 mg/kg/d gradually reduced the seizure frequency. The patient attained a status-free condition and approximately 50% reduction in seizure frequency, resulting in decreased hospital admissions and costs [32].

Another case of EIEE linked to biallelic *SCN1B* variants was reported by Aeby et al. [33]. The girl had focal and focal-to-bilateral clonic seizures and refractory myoclonic SE since the age of 3 months, not responding to ASMs such as levetiracetam, valproate, topiramate, and clobazam or a ketogenic diet. By the age of 28 months, treatment with FFA (0.6 mg/kg/d) was started and resulted in a significant reduction in seizure frequency and full resolution of recurrent SE episodes for over 2 years of follow-up [33].

Overall, SE and the sudden unexpected death in epilepsy (SUDEP) are the leading seizure-related causes of death in patients with epilepsy and DEEs such as DS [34, 35] and LGS [36]. A recent pooled analysis of phase III clinical trials and long-term open-label observational studies showed a reduction of all-cause (including SE-related) and SUDEP mortality rates in DS patients treated with FFA [37]. Cautiously, the use of FFA might be assumed to potentially lead to a reduction in mortality. However, a limitation of our review is that the collected cases may be subject to publication bias since successful cases are more likely to be reported.

## Conclusions

Based on the four case reports of acute SE treatment with FFA, and reduced rates of SE during FFA treatment in observational studies and case reports, FFA may prove useful in treating and preventing SE. Treatments that suppress refractory SE are critically needed. Further studies should investigate whether earlier treatment with FFA leads to better and earlier control of SE.

## Data Availability

The datasets used and analyzed during the current study are available from the corresponding author on reasonable request.

## Abbreviations

ASM: Antiseizure medication
CARE: CAse Reports guidelines
CCI: Charlson Comorbidity Index
MRI: Magnetic resonance imaging
EEG: Electroencephalography
ILAE: International League Against Epilepsy
mRS: Modified Rankin Scale
NCSE: Non-convulsive status epilepticus
SD: Standard deviation
SE: Status epilepticus
STESS: Status Epilepticus Severity Score
